# Prognostic significance of anti-diabetic medications in pancreatic cancer: A meta-analysis

**DOI:** 10.18632/oncotarget.17728

**Published:** 2017-05-09

**Authors:** Dong-Chu Zhou, Hui Gong, Chong-Qing Tan, Jian-Quan Luo

**Affiliations:** ^1^ Department of Pharmacy, The Second Xiangya Hospital, Central South University, Changsha, Hunan 410011, China; ^2^ Institute of Clinical Pharmacy, Central South University, Changsha, Hunan 410011, China

**Keywords:** anti-diabetic medications, pancreatic cancer, prognosis, mortality, meta-analysis

## Abstract

The role of anti-diabetic medications in pancreatic cancer remains conflicting. We carried out a systematic search of Pubmed and Embase databases for studies published before August 2016, which assessed the associations between anti-diabetic medications (metformin, sulfonylureas, thiazolidinediones and insulin) intake and pancreatic cancer prognosis. Hazard ratios (HRs) with 95% confidence intervals (CIs) were estimated using the random-effects model. The primary outcomes of interest were overall survival (OS) and progression-free survival (PFS). Fourteen studies enrolling 94778 participants were eligible for inclusion, with 12 cohort studies and 2 randomized controlled trials (RCTs). Significant association between metformin (adjusted HR=0.77, 95% CI=0.68-0.87) use and OS was found in cohort studies, whereas no significant association between metformin use and PFS (HR=1.22; 95% CI=0.76-1.95) or OS (HR=1.20, 95% CI=0.84-1.72) in RCTs. No significant survival benefits were identified for insulin (HR=1.18, 95% CI=0.83-1.69), sulfonylureas (HR=1.03, 95% CI=0.81-1.30), or thiazolidinediones (HR=0.84, 95% CI=0.58-1.22). The trim-and-fill method and subgroup analyses stratified by the study characteristics confirmed the robustness of the results. Our findings provide strong evidence that metformin is associated with improved OS in pancreatic cancer patients in cohort studies. However, the effect of other anti-diabetic medications should be interpreted with caution owing to the limited number of studies.

## INTRODUCTION

Pancreatic cancer (PC) is the fourth leading cause of cancer-related death in the United States [1]. Owing to late stage at the time of diagnosis, there are just 10%-20% of patients eligible for surgical treatment [2]. Although the surgical procedure of pancreatic cancer over the last decades has improved strongly, it needs some more effective treatments and adjuvant therapies against PC.

The relationship between PC and diabetes mellitus (DM) has been increasingly recognized over the past decades. Studies suggest that DM plays a pivotal role in cancer risk and progression [3–5]. Although we have not fully understood the mechanisms of increased risk of cancer incidence with DM, hyperinsulinemia may influence the neoplastic process through its effects on enhancing cancer cell proliferation, survival, and invasion and inhibiting apoptosis in the insulin-like growth factor-I (IGF-I) signaling pathway [6, 7]. There are an increasing number of experimental evidence and epidemiologic studies to show that ADMs may modify the prognosis of PC. Some studies suggest that metformin may improve outcome of patients with diabetes and pancreatic cancer [8–13], whereas others have not revealed beneficial effect [14–18]. Besides, some studies suggest that insulin may highlight risk of PC mortality [19, 20], whereas others have not affected the survival [8, 11]. Due to controversial results among studies, we thus carry out this meta-analysis to investigate the prognostic value of ADMs use (as compared with non-user) among PC patients.

## RESULTS

### Description of the included studies

**Figure 1:**
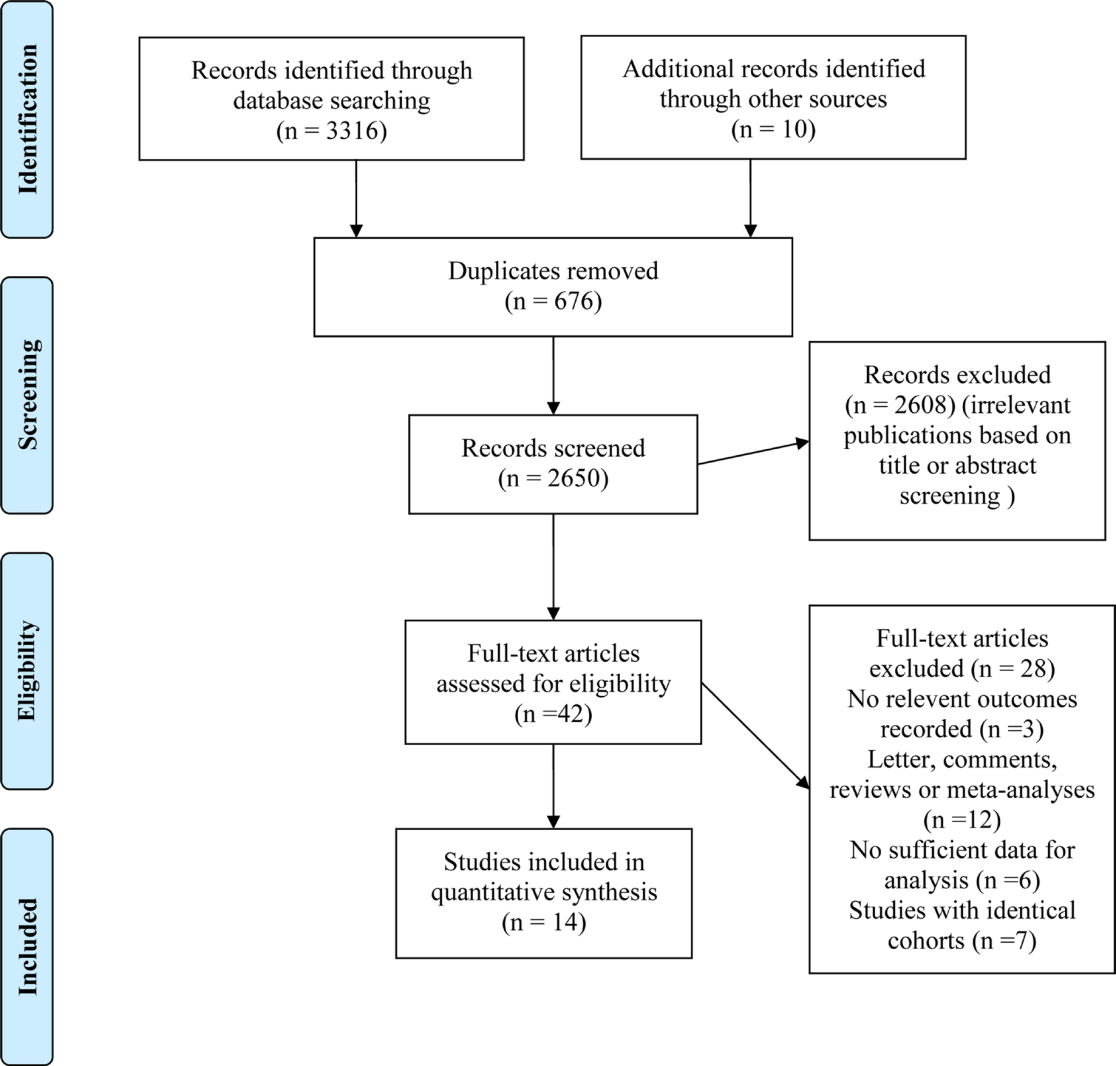
Flow diagram of the selection process of studies investigating effect of anti-diabetic medications use on pancreatic cancer survival

The initial database search yielded a total of 3326 references for eligibility. After excluding the duplicates and screening the remaining title and abstract, 42 potentially relevant studies were identified for further review. After selection, a total of 14 publications met our inclusion criteria (Figure 1). The clinical features of included studies were summarized in Table 1. In summary, 13 studies investigated the survival outcomes for patients of metformin use, 5 for insulin use, 2 for SUs use and 2 for TZDs use. The median follow-up time ranged from 0.77 to 12 years. 5 studies were carried out in USA, 3 in Europe and 2 in Asia. Several cohorts were adjusted for some conventional influential factors, including age, sex, disease stage. Six studies involved PC patients with I-IV disease stages, and two with stage IV. Assessment of methodological quality for cohort studies yielded a score range of 7 to 9, and 7 of 12 studies had a score of 8 or above (Supplementary Table 1).

**Table 1:** Baseline characteristics of the included studies on survival outcomes of anti-diabetic medications use for pancreatic cancer patients

Authors	Study design	Country/Setting	No. of hospitals involved	Study period	Exposure ascertainment	Median follow-up (months)	ADMs user/non-user	Sample size	Types of ADMs	Disease stage	Survival end points	Adjusted variables
Reni et al.	RCT	Italy; hospital based	Single	2010-2014	RCT	NR	31/29	60	Metformin	IV	OS, PFS	NR
Lee et al.	Retrospective cohort	Korea; hospital based	Single	2005-2013	Medical records, self-reported	10.3	117/120	237	Metformin TZDs SU insulin	I-IV	OS	CA199 levels, tumor size and stage, performance status, DDP4 inhibitors
Kozak et al.	Retrospective cohort	USA; hospital based	Single	1998-2013	Electronic medical records	11.23	18/153	171	Metformin	I-IV	OS, DFS	Age, sex, smoking status, surgery, tumor stage, treatment regimen
Choi et al.	Retrospective cohort	Korea; hospital based	Single	2003-2010	Electronic medical records	10.2	56/127	183	Metformin SU insulin	I-IV	OS	Performance status, diabetes, cancer extent, weight loss during therapy
Chaiteerakij et al.	Retrospective cohort	USA; hospital based	Single	2000-2011	Electronic medical records	9.26	366/614	980	Metformin	I-IV	OS	Age, sex, BMI, stage
Cerullo et al.	Retrospective cohort	USA; population based	Multiple	2010-2012	Electronic medical records	16.5	456/2937	3393	Metformin	I-IV	OS	Age, sex, region, Charlson index, treatment regimen
Ambe et al.	Prospective cohort	USA; hospital based	Single	1986-2013	Electronic medical records	19	19/25	44	Metformin	I-II	OS	Age, BMI, surgery, diabetes, CA199 levels, stage, regional nodes
Kordes et al.	RCT	Netherlands; hospital based	Multiple	2010-2014	RCT	28.1	61/60	121	Metformin	IV	OS, PFS	Age, sex, performance status, stage, tumor location, surgery, diabetes
Tseng et al.	Retrospective cohort	China; population based	Multiple	1995-2006	Structured questionnaire interview	12 years	5927/80970	86897	Insulin	I-IV	OS	Age, sex, diabetes, BMI, smoking, region
Hwang et al.	Retrospective cohort	United Kingdom; population based	Multiple	2003-2010	Electronic medical records	NR	247/269	516	Metformin	I-IV	OS	Age, sex, diabetes duration and complications, Charlson index, BMI, GFR, smoking, other ADMs and HbA1c.
Sadeghi et al.	Retrospective cohort	USA; hospital based	Single	2000-2009	Interviews, medical records.	11.4	117/185	302	Metformin	I-IV	OS	Disease stage, CA199 level, tumor size and site, performance status
Amin et al.	Retrospective cohort	USA; population based	Multiple	2007-2011	NR	NR	589/258	847	Metformin	I-IV	OS	Demographic factors, stage, income, diabetic complications, Charlson index, other ADMs
Jang et al.	Prospective cohort	Korea; population based	Multiple	2005-2011	Prescription information	NR	530/234	764	Metformin	I-IV	OS	NR
Jeon et al.	Retrospective cohort	USA; population based	Multiple	2008-2009	NR	NR	132/131	263	Insulin/SUs; metformin/TZDs	I-IV	OS	Age, sex, race, stage and chemotherapy

ADMs, anti-diabetic medications; BMI, body mass index; DFS, disease-free survival; NR, not reported; OS, overall survival; PFS, progression-free survival; RCT, randomized controlled trial; SUs, sulfonylureas; TZDs, thiazolidinediones.

### Metformin use and PC survival

**Figure 2:**
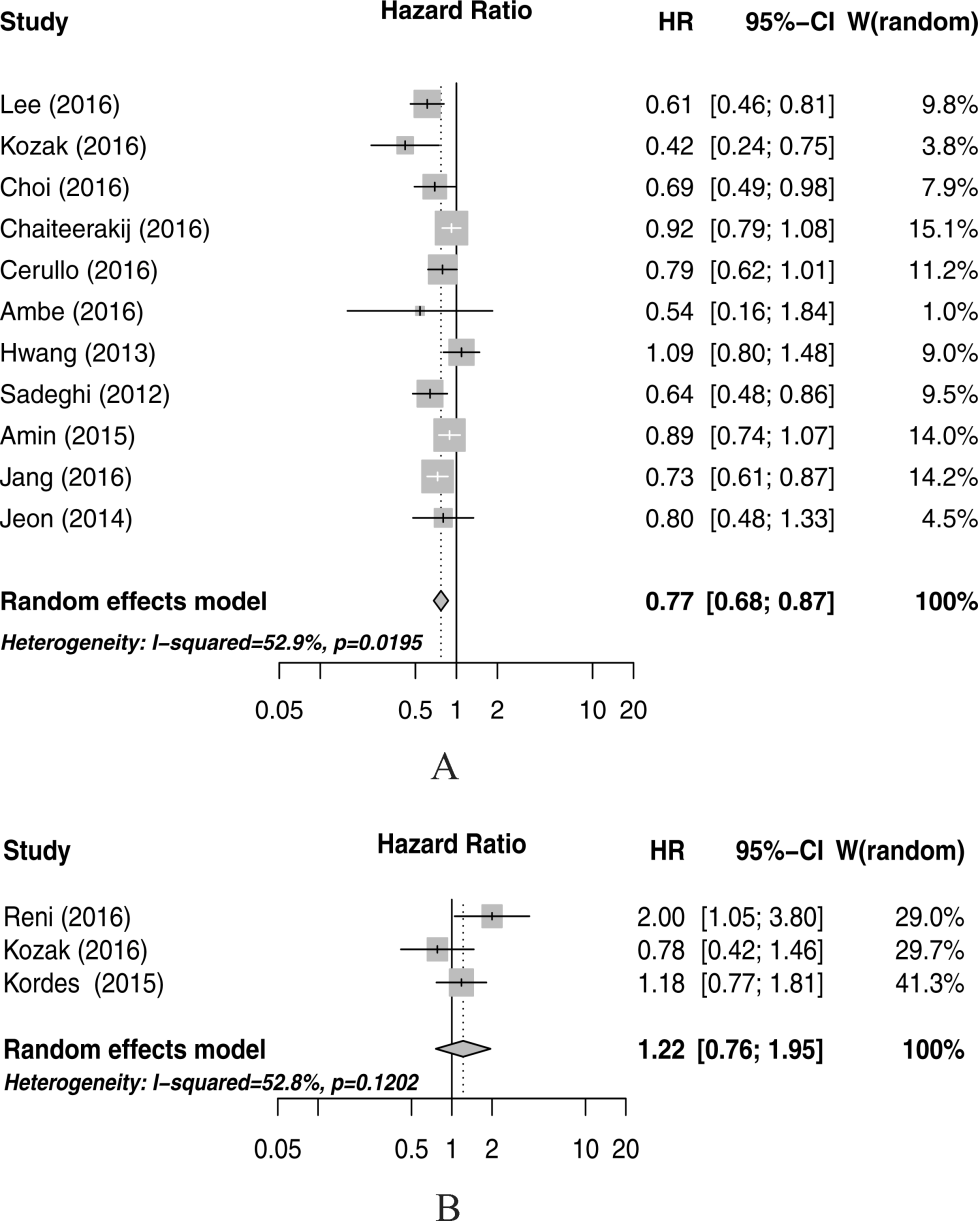
Meta-analysis of the associations between metformin use and pancreatic cancer overall survival (A), and progression-free surival (B) CI, confidence interval; HR, hazard ratio; W (random): Weights (random effects model).

The combined HR for the OS comparing metformin use versus non-use was 0.77 (95% CI=0.68-0.87) with moderate inter-study heterogeneity (I^2^=52.9%, p=0.02) (Figure 2A) for cohort studies. Figure 2B presents the HR (HR=1.22; 95% CI=0.76-1.95) for PFS. No significant survival benefit was noted for randomized controlled trials (RCTs) (HR=1.20, 95% CI=0.84-1.72).

We summarized the subgroup analyses for OS in Table 2 to further test potential sources of heterogeneity among certain major clinical characteristics of the included studies. The pooled HRs for the majority of the subgroups did not change significantly stratified by some major study characteristics, including the study design and setting, study country, the number of hospitals, sample size, adjusted variables or the scores of Newcastle–Ottawa Quality Assessment Scale. A possible interaction was found in the subgroup of sample size. Results of analyses limited to studies with some of the main variable adjusted (age, sex and tumor stage) are also presented in Table 2. For studies with these three variables adjusted, a null prognostic association of metformin use was noted. Nevertheless, further studies should be conducted to examine the true survival benefit of metformin in PC patients due to the small number of studies involved in these subgroups.

**Table 2:** Subgroup analyses of the associations between metformin use and overall survival for cohort studies

Comparison variables	Overall survival			
	No. of studies	I^2^ statistics; %	HR (95% CI)	*P*_interation_
Total	11	52.9	0.77(0.68 - 0.87)	NA
Study design				0.534
Prospective cohort	2	0	0.72(0.61 - 0.86)	
Retrospective cohort	9	58.9	0.78(0.67 -0.90)	
Study setting				0.111
Hospital based	6	64.5	0.67(0.53 - 0.85)	
Population based	5	33.6	0.84(0.73 - 0.96)	
Study region				0.214
USA	7	47.2	0.78(0.67 - 0.92)	
Europe	1		1.09(0.80 - 1.48)	
Asia	3	0	0.69(0.60 - 0.79)	
Hospital number				0.111
Single	6	64.5	0.67(0.53 - 0.85)	
Multiple	5	33.6	0.84(0.73 -0.96)	
Sample size				0.0024
≥500	5	44.1	0.86(0.76 -0.97)	
<500	6	0	0.63(0.54 - 0.74)	
Main variable adjusted*				0.276
Yes	5	57.8	0.83(0.68 -1.03)	
No	6	30.8	0.73(0.64 -0.83)	
NOS scale				0.359
≥8	6	59.4	0.73(0.59 - 0.90)	
<8	5	36.2	0.82(0.71 - 0.94)	

CI, confidence interval; HR, hazard ratio; Main variable adjusted*, Age, sex, stage; NA; not available.

**Figure 3:**
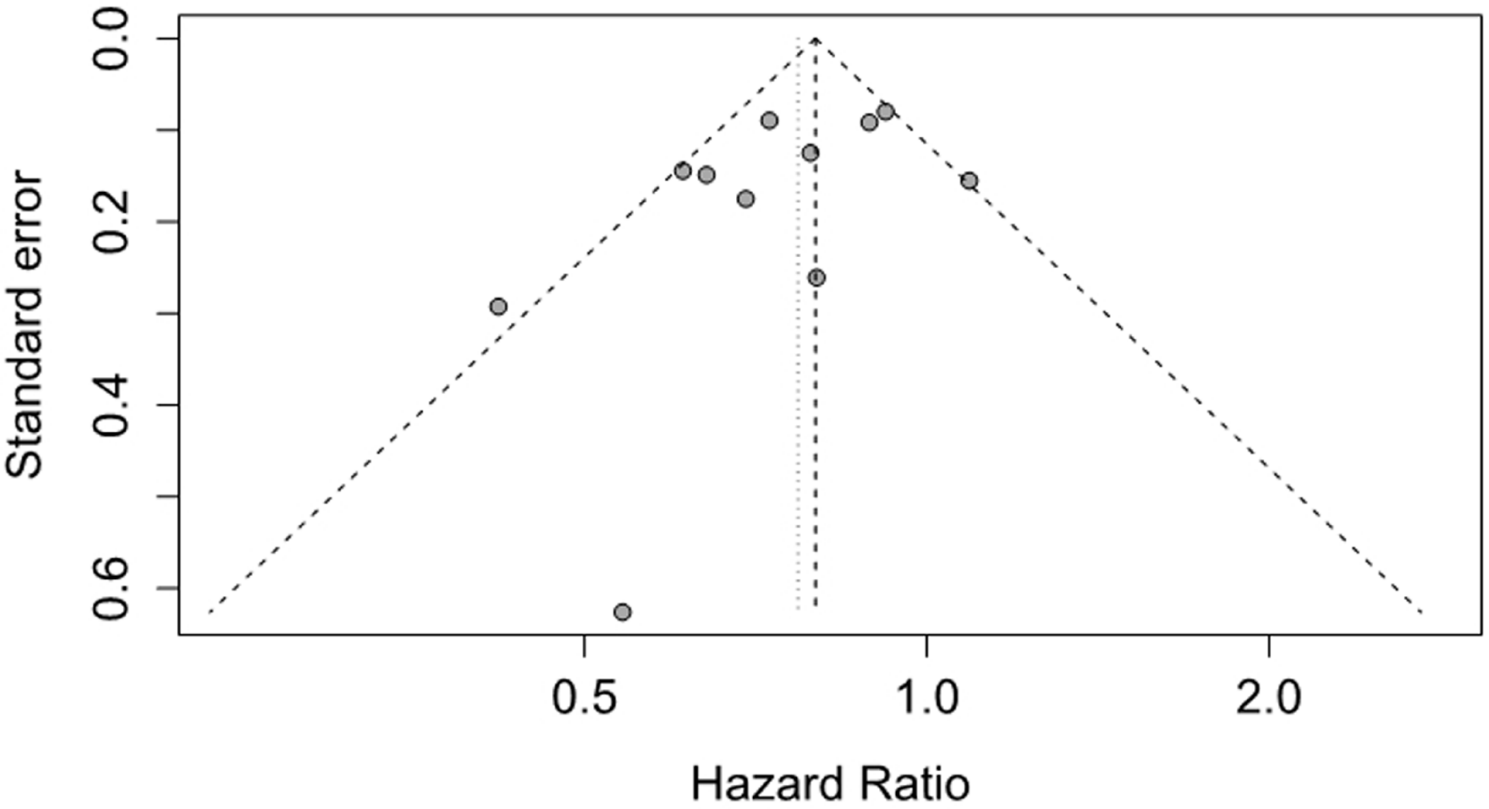
Funnel plot of studies investigating association between metformin use and pancreatic cancer survival for cohort studies

Sensitivity analysis by omitting one single study each time and pooling the others indicated that the pooled HRs was not significantly altered. Funnel plot for publication bias did not show asymmetry (Figure 3). Further Egger’s test (*P*=0.135) or Begg’s test (*P*=0.436) also did not found a certain degree publication bias.

### Other ADMs use and PC survival

**Figure 4:**
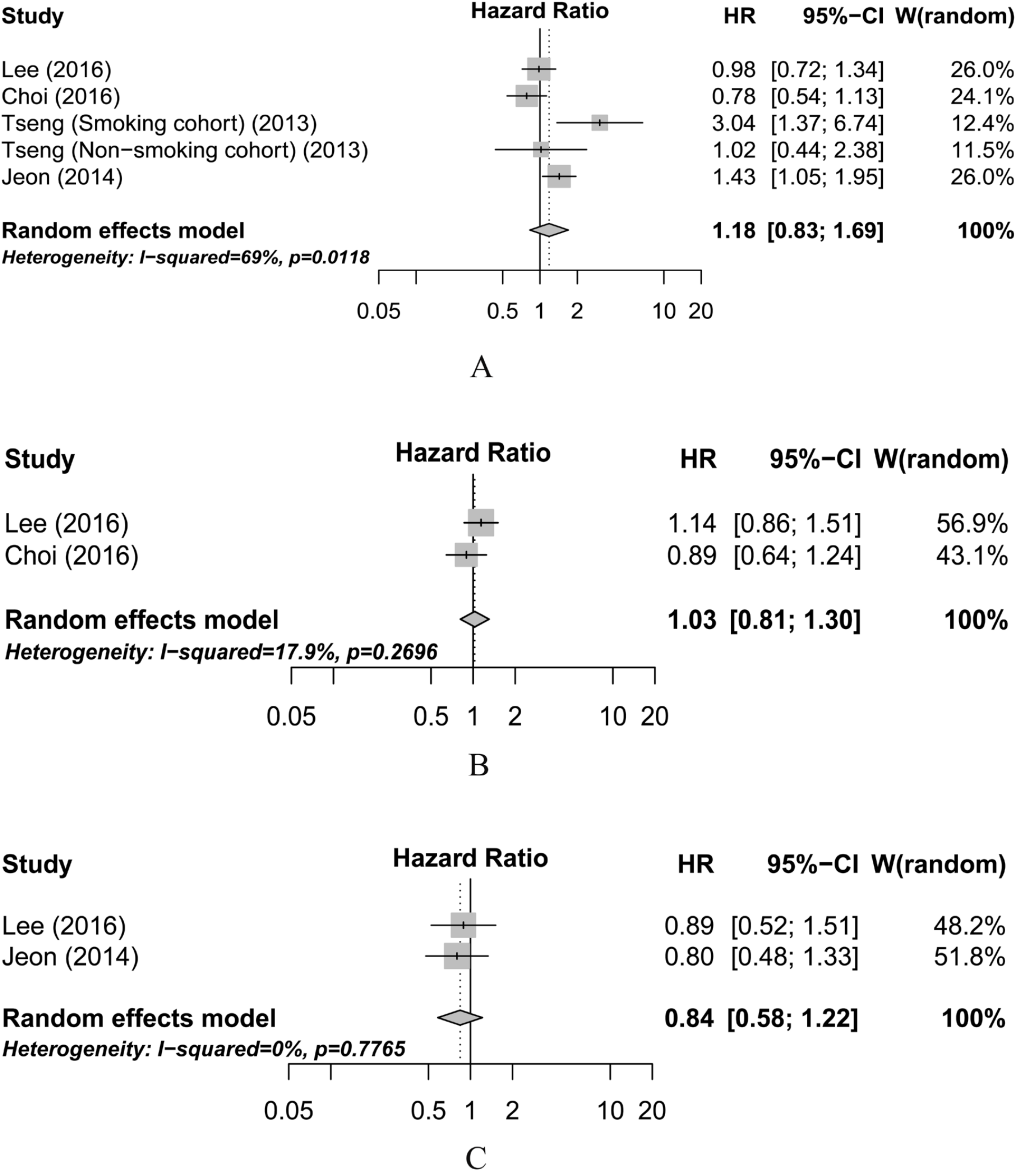
Meta-analysis of the associations between insulin (A), sulfonylureas (B), and thiazolidinediones (C) use and pancreatic cancer overall survival CI, confidence interval; HR, hazard ratio; W (random): Weights (random effects model).

Five studies investigated the impact of insulin use and PC survival and there was no significant association between insulin use and PC survival (HR=1.18, 95% CI=0.83-1.69; Figure 4A). We also did not find significant association between SUs (HR=1.03, 95% CI=0.81-1.30; Figure 4B) or TZDs (HR=0.84, 95% CI=0.58-1.22; Figure 4C) use and PC survival.

## DISCUSSION

This meta-analysis investigated the association between ADMs (metformin, insulin, SUs and TZDs) treatment and survival of PC. We found metformin treatment was significantly associated with favorable OS of PC patients (HR=0.77, 95% CI=0.68-0.87) in cohort studies, but was not significantly associated with PFS (HR=1.22; 95% CI=0.76-1.95) for RCTs. We also found no survival benefits of other ADMs, such as insulin, SUs or TZDs, for PC patients.

Several potential mechanisms may explain the associations for the fact that conventional ADMs may alter the risk of multiple malignancies. It was reported that metformin has been shown to play an important anticancer role in multiple ways including insulin-dependent or independent manners [21]. A recent study found that SUs can induce cell proliferation and had an effect of carcinogenesis by promoting insulin secretion [22]. Moreover, previous *in vitro* studies showed that TZDs had an impact on cell growth arrest and apoptosis and the inhibition of cancer cell invasion [23].

Several important strengths of this meta-analysis should be addressed. Firstly, to the best of our knowledge, this is the first systematic review regarding the associations between the use of ADMs and prognosis of PC. Secondly, comprehensive and reproducible search strategies were developed to identify all relevant studies or trials in the major databases without language limitations. Thirdly, we investigated the most commonly used ADMs including metformin, SUs, TZDs and insulin and conducted a meta-analysis for both RCTs and cohort studies. Fourthly, more than 90000 participants were included to quantitatively assess the association between ADMs use and PC prognosis, which was the most comprehensive synthesis of the evidence on this topic ever today. Finally, several subgroup analyses were carried out for some of the important variables, such as study design and setting, research region, number of research hospital, main variable adjusted and quality score. The results showed consistency across subgroups.

Still there are limitations in our systematic review. Firstly, the number of studies for each medication involved in this meta-analysis was relatively small except for metformin, and thus it is difficult to draw definite conclusions for the limited statistical power in SUs, TZDs or insulin subset. Secondly, almost none of the included studies had dose or duration-response analysis for certain ADMs, so it is impossible for us to perform this kind of analysis. Therefore, further study should be focused on this aspect. Thirdly, although some major confounders including age, sex and disease stage were identified and adjusted for some of the included studies, some other variables (such as tumor size, body mass index or chemotherapy) could influence our exploration of associations between ADMs and PC survival. Last but not the least, although we did not find significant publication bias for metformin subset in cohort studies, we could not totally exclude potential impact of unpublished studies on the pooled results, which might have resulted in reporting bias. However, the adjusted estimates of the results using the trim and fill methods remained unchanged, indicating the stability of our analysis.

In summary, the results from this meta-analysis revealed that in cohort studies, metformin, not other ADMs was associated with improved OS in PC patients. However, due to limited number of studies investigating other ADMs, further large-scale studies are warranted to determine these associations.

## MATERIALS AND METHODS

### Literature search and study selection

Based on the PRISMA statement [24], we performed a comprehensive literature search in Pubmed and Embase databases up to August 2016 for relevant citations without language restrictions. We used the search strategies (Supplementary Table 2 and Table 3) that included Medical Subject Headings and Emtree headings combined key words relating to the prognostic effect of ADMs among PC patients. We also manually scanned the reference lists from the extracted relevant research papers, previous reviews and meta-analysis for additional possible publications.

We included published studies providing aggregate data if they met the following criteria: (1) evaluated any prognostic information in PC patients comparing ADMs users with non-users, (2) reported a summary statistic of hazard ratios (HRs) with 95% confidence intervals (CIs) or provided date for calculation as described by Parmar et al [25]. RCTs or observational studies were eligible for this meta-analysis. If there were more than one studies from the same cohort, we selected the most detailed or recent one for analysis. All the studies reporting prognostic information, including overall survival (OS), and progression-free survival (PFS), were selected in the main analyses (Supplementary Table 4). Two independent investigators (Zhou and Gong) conducted the study selection from eligible studies.

### Data extraction

Two independent investigators (Zhou and Gong) selected articles and extracted data from eligible studies, evaluated the quality of each study and any discrepancies were resolved by a consensus discussion with a third investigator (Tan). The characteristics recorded were the first author’s last name, publication year, country of the population studied, study design, study setting, number of hospitals involved, time period of study, information source for exposure ascertainment and outcome assessment, total number of persons in each group (exposed vs. not exposed), sample size, types of ADMs, stage, mean F/U (months), survival endpoints and adjustment variables HR, and 95% confidence intervals (CIs) with adjustment for confounding factors. The methodological quality of each study was evaluated using the Newcastle-Ottawa quality assessment scale [26], in which three domains including cohort selection, comparability, and outcome were evaluated with a score range of 0 to 9 with nine representing the highest quality.

### Statistical analysis

We used STATA statistical software (version 12.0, StataCorp LP, College Station, TX) and R statistical software (version 3.3.1) to perform the meta-analysis. Survival estimates with full adjustments for known confounders of included studies were abstracted. Summary data reporting HRs with corresponding 95% CIs estimated from Cox proportional hazards models were pooled with random-effects model [27]. The data regarding the association of ADMs (use vs. no use) with survival outcomes were pooled separately. We used the Cochrane Q statistic (with a P value less than 0.10 considering statistically significant) and the *I*^*2*^ statistic (with an *I*^*2*^ exceeding 50% indicating significant heterogeneity) to test for between-study heterogeneity [28]. Metformin usage and OS for PC patients were explored for primary meta-analysis. Other outcome measures such as PFS and Disease-free survival (DFS) were also evaluated. Owing to the limited studies for PFS and DFS, we combined the data of PFS and DFS as one outcome for the meta-analysis. We performed sensitivity analyses to explore the reasons for statistical heterogeneity. The risk of publication bias was assessed visually by inspecting of a funnel plot and statistically by using Egger's or Begg's regression model [29]. We further ascertained the number of missing studies using Duval and Tweedie’s trim and fill method to adjust the summary hazard ratio based on all the studies including the hypothesized missing ones [30]. All statistical analyses were two-sided and a P-value less than 0.05 was considered significant.

## SUPPLEMENTARY MATERIALS TABLES



## Abbreviations

ADMs: anti-diabetic medications
SUs: sulfonylureas
TZDs: thiazolidinediones
DM: diabetes mellitus
PC: Pancreatic cancer
HRs: hazard ratios
CIs: confidence intervals
OS: overall survival
PFS: progression-free survival
RCTs: randomized controlled trials
