# Supplementary material for: Prognostic significance of anti-diabetic medications in pancreatic cancer: A meta-analysis
Source: Oncotarget. 2017 May 9;8(37):62349–57. doi: 10.18632/oncotarget.17728 (PMC5617510; doi:10.18632/oncotarget.17728)
Supplement: Supplementary file 1 [file oncotarget-08-62349-s001.pdf]

Prognostic significance of anti-diabetic medications in pancreatic cancer: A meta-analysis

SUPPLEMENTARY MATERIALS

REFERENCES

1. Reni M, Dugnani E, Cereda S, Belli C, Balzano G, Nicoletti R, Liberati D, Pasquale V, Scavini M, Maggiora P, Sordi V, Lampasona V, Ceraulo D, et al. (Ir)relevance of Metformin Treatment in Patients with Metastatic Pancreatic Cancer: An Open-Label, Randomized Phase II Trial. Clin Cancer Res. 2016; 22:1076–85.
2. Lee SH, Yoon SH, Lee HS, Chung MJ, Park JY, Park SW, Song SY, Chung JB, Bang S. Can metformin change the prognosis of pancreatic cancer? Retrospective study for pancreatic cancer patients with pre-existing diabetes mellitus type 2. Digestive and liver disease. 2016; 48:435-440.
3. Kozak MM, Anderson EM, von Eyben R, Pai JS, Poultsides GA, Visser BC, Norton JA, Koong AC, Chang DT. Statin and Metformin Use Prolongs Survival in Patients With Resectable Pancreatic Cancer. Pancreas. 2016; 45:64-70.
4. Jang WI, Kim MS, Kang SH, Jo AJ, Kim YJ, Tchoe HJ, Park CM, Kim HJ, Choi JA, Choi HJ, Paik EK, Seo YS, Yoo HJ, et al. Association between metformin use and mortality in patients receiving curative resection for pancreatic cancer: A nationwide population-based study. Liver Cancer. 2016; 5:75.
5. Choi Y, Kim TY, Oh DY, Lee KH, Han SW, Im SA, Kim TY, Bang YJ. The Impact of Diabetes Mellitus and Metformin Treatment on Survival of Patients with Advanced Pancreatic Cancer Undergoing Chemotherapy. Cancer research and treatment. 2016; 48:171-179.
6. Chaiteerakij R, Petersen GM, Bamlet WR, Chaffee KG, Zhen DB, Burch PA, Leof ER, Roberts LR, Oberg AL. Metformin Use and Survival of Patients With Pancreatic Cancer: A Cautionary Lesson. Journal of clinical oncology. 2016; 34:1898-1904.
7. Cerullo M, Gani F, Chen SY, Canner J, Pawlik TM. Metformin Use Is Associated with Improved Survival in Patients Undergoing Resection for Pancreatic Cancer. Journal of gastrointestinal surgery. 2016; 20:1572-1580.
8. Ambe CM, Mahipal A, Fulp J, Chen L, Malafa MP. Effect of Metformin Use on Survival in Resectable Pancreatic Cancer: A Single-Institution Experience and Review of the Literature. PloS one. 2016; 11:e0151632.
9. Kordes S, Pollak MN, Zwinderman AH, Mathot RA, Weterman MJ, Beeker A, Punt CJ, Richel DJ, Wilmink JW. Metformin in patients with advanced pancreatic cancer: a double-blind, randomised, placebo-controlled phase 2 trial. American journal of physiology Regulatory, integrative and comparative physiology. 2015; 16:839-847.
10. Amin S, Mhango G, Lin J, Boffetta P, Wisnivesky J, Lucas A. Metformin use does not improve survival among diabetics with pancreatic adenocarcinoma: A population-based analysis. American Journal of Gastroenterology. 2015; 110:S17.
11. Jeon CY, Pandol SJ, Goodman MT. Survival time in pancreatic cancer patients with metabolic syndrome varies by use of insulin and statins. Cancer research. 2014; 74:2173.
12. Tseng CH. Diabetes, insulin use, smoking, and pancreatic cancer mortality in Taiwan. Acta diabetologica. 2013; 50:879-886.
13. Hwang AL, Haynes K, Hwang WT, Yang YX. Metformin and survival in pancreatic cancer: a retrospective cohort study. Pancreas. 2013; 42:1054-1059.
14. Sadeghi N, Abbruzzese JL, Yeung SC, Hassan M, Li D. Metformin use is associated with better survival of diabetic patients with pancreatic cancer. Clinical cancer research. 2012; 18:2905-2912.
15. Cerullo M, Gani F, Canner JK, Pawlik TM. Improved survival following pancreatic cancer among patients receiving metformin. Gastroenterology. 2016; 150:S1202.
16. Jo A, Kim Y, Kang S, Kim M, Ko M. The Effect Of Metformin Use And Mortality Among Those With Pancreatic Cancer And Type 2 Diabetes Mellitus: Findings From A Nationwide Population Retrospective Cohort Study. Value in health. 2015; 18:A439.
17. Chaiteerakij R, Zhen DB, Burch PA, Chaffee KG, Bamlet WR, Oberg AL, Roberts LR, Petersen GM. Metformin use does not increase survival of pancreatic cancer patients: A cautionary lesson. Cancer research. 2015; 75:S15.
18. Ambe C, Mahipal A, Fulp WJ, Chen DT, Malafa MP. Effect of metformin use on the survival outcomes in diabetic patients with resectable pancreatic cancer: A single-institutional experience and meta-analysis. Journal of Clinical Oncology. 2015; 33:S3.
19. Oh DY, Choi Y, Kim TY, Lee KH, Han SW, Im SA, Kim TY, Bang YJ. The impact of diabetes mellitus and metformin on survival of patients with advanced pancreatic cancer receiving chemotherapy. Journal of Clinical Oncology. 2013; 31:S15.

20. Hwang A, Haynes K, Hwang WT, Yang YX. Metformin and survival in pancreatic cancer: A retrospective cohort study. American Journal of Gastroenterology. 2012; 107:S84.
21. Sadeghi N, Abbruzzese JL, Yeung SJ, Hassan M, Li D. Effect of metformin on survival of diabetic patients with pancreatic adenocarcinoma. Journal of Clinical Oncology. 2011; 29:S15.
22. Jeon A, Pandharipande PV, Kong CY, Hur C. Metformin chemoprevention against pancreatic adenocarcinoma in patients with type 2 diabetes mellitus: Results of a disease simulation model. Value in Health. 2016; 19:A137.
23. Funch D, Gydesen H, Tornoe K, Major-Pedersen A, Chan KA. A prospective, claims-based assessment of the risk of pancreatitis and pancreatic cancer with liraglutide compared to other antidiabetic drugs. Diabetes, obesity & metabolism. 2014; 16:273-275.
24. Lee MS, Hsu CC, Wahlqvist ML, Tsai HN, Chang YH, Huang YC. Type 2 diabetes increases and metformin reduces total, colorectal, liver and pancreatic cancer incidences in Taiwanese: a representative population prospective cohort study of 800,000 individuals. BMC cancer. 2011; 11:20.
25. Yang YX, Rustgi AK. Impact of Metformin on Advanced Pancreatic Cancer Survival: Too Little, Too Late? Clinical cancer research. 2016; 22:1031-1033.
26. De Souza A, Khawaja KI, Masud F, Saif MW. Metformin and pancreatic cancer: Is there a role? Cancer chemotherapy and pharmacology. 2016; 77:235-242.
27. Bhaw-Luximon A, Jhurry D. Metformin in pancreatic cancer treatment: from clinical trials through basic research to biomarker quantification. Journal of cancer research and clinical oncology. 2016.
28. Zhang JW, Sun Q. Metformin may improve the prognosis of patients with pancreatic cancer. Asian Pacific journal of cancer prevention. 2015; 16:3937-3940.
29. Wendling P. AACR: Metformin survival benefit shaky in pancreatic cancer. Oncology Report. 2015; 11:24.
30. Aung KL, Moore MJ. Metformin for pancreatic cancer. The Lancet Oncology. 2015; 16:748-749.
31. Wang Z, Lai ST, Xie L, Zhao JD, Ma NY, Zhu J, Ren ZG, Jiang GL. Metformin is associated with reduced risk of pancreatic cancer in patients with type 2 diabetes mellitus: a systematic review and meta-analysis. Diabetes research and clinical practice. 2014; 106:19-26.
32. Liu Y. Metformin and pancreatic cancer risk in type 2 diabetic patients: A systematic review and meta-analysis. Pharmacotherapy. 2014; 34:e296.
33. Gong J, Robbins LA, Lugea A, Waldron RT, Jeon CY, Pandol SJ. Diabetes, pancreatic cancer, and metformin therapy. Frontiers in physiology. 2014; 5:426.
34. Burney S, Khawaja KI, Saif MW, Masud F. Chemotherapy and metformin in pancreatic adenocarcinoma and neuroendocrine tumors. JOP. 2014; 15:313-316.
35. Singh PP, Singh S, Singh A, McWilliams R, Chari S. Metformin and risk of pancreatic cancer in patients with diabetes mellitus: A systematic review and meta-analysis. American Journal of Gastroenterology. 2012; 107:S96.
36. Pollak M. Metformin and pancreatic cancer: A clue requiring investigation. Clinical Cancer Research. 2012; 18:2723-2725.
37. Yoon SH, Lee HS, Chung MJ, Park JY, Park SW, Song SY, Chung JB, Bang S. Can metformin change the prognosis of pancreatic cancer? Gastroenterology. 2015; 148:S941.
38. Wilmink J, Kordes S, Zwinderman K, Mathot R, Punt CJA, Richel D. A phase II randomized, placebo controlled study to evaluate the efficacy of the combination of gemcitabine, erlotinib, and metformin in patients with locally advanced or metastatic pancreatic cancer. Journal of Clinical Oncology. 2014; 32:4021.
39. Esbah O, Eren T, Helvaci K, Guler T, Duran AO, Bal O, Özdemir NY, Oksuzoglu B, Aksoy S. The effect of metformin use on prognosis in pancreatic cancer patients. Acta Medica Mediterranea. 2014; 30:465-471.
40. Toomey P, Ross S, Joseph A, Teta A, Paul H, Luberice K, Cohen K, Rosemurgy A. Metformin does not increase survival for patients with diabetes who undergo pancreaticoduodenectomy for pancreatic adenocarcinoma. HPB. 2013; 15:77-78.
41. Esbah O, Oksuzoglu B, Eren T, Helvaci K, Guler T, Duran AO, Bal O, Ozdemir NY, Aksoy S. Metformin in diabetic pancreatic cancer patients: Benefit or not-Multicenter experience. Journal of Clinical Oncology. 2013; 31:e15110.
42. Witkowski ER, Ragulin-Coyne E, Chau Z, McDade TP, Ng SC, Tseng JF. Effects of metformin on pancreatic cancer survival among medicare beneficiaries. Journal of Clinical Oncology. 2012; 30:e14691.

Supplementary Table 1: Methodological quality of the included studies based on the Newcastle–Ottawa Scale

	Observational studies[a]				
	**Design**	**Selection**	**Comparability**	**Outcome/exposure**	**Overall quality (max 9)**
Lee *et al.*(2016)	Cohort	***	**	***	8
Kozak *et al.*(2016)	Cohort	***	**	***	8
Choi *et al.* (2016)	Cohort	***	**	**	7
Chaiteerakij *et al.*(2016)	Cohort	***	**	**	7
Cerullo *et al.*(2016)	Cohort	****	**	***	9
Ambe *et al.*(2016)	Cohort	***	**	***	8
Tseng *et al.*(2013)	Cohort	****	**	***	9
Hwang *et al.*(2013)	Cohort	****	**	**	8
Sadeghi *et al.*(2012)	Cohort	***	**	**	7
Amin *et al.*(2015)	Cohort	***	**	**	7
Jang *et al.*(2016)	Cohort	****	**	**	8
Jeon *et al.* (2014)	Cohort	***	**	**	7

	Randomized controlled trials[b]							
	1	2	3	4	5	6	7	
Reni *et al.*(2016)	L	L	L	L	H	U	L	NA
Kordes *et al.*(2015)	L	L	L	L	H	U	L	NA

[a] Study quality assessment of observational studies performed using the Newcastle–Ottawa scale (each asterisk represents if individual criterion within the subsection were fulfilled).

[b] Cochrane Collaboration's tool for assessing risk of bias for randomized trials, which assesses the risk of bias (L=low, H=high, U=unclear) across 7 domains:1 (Random sequence generation), 2 (Allocation concealment), 3 (Blinding of participants and researchers), 4 (Blinding of outcome assessment), 5 (Incomplete outcome data), 6 (Selective reporting) and 7 (Other bias). NA, not available

Supplementary Table 2: Search strategy for Pubmed (Publication date to 2016/08/17)

1. "Pancreatic Neoplasms"[Mesh]
2. Pancreatic[Title/Abstract] OR Pancreas[Title/Abstract]
3. (Cancer OR tumor OR tumor OR carcinoma OR neoplastic* OR malignant*)[Title/Abstract]
4. 2 AND 3
5. 1 OR 4
6. "Hypoglycemic Agents"[Mesh]
7. Hypoglycemic*[Title/Abstract] OR antihyperglycemic *[Title/Abstract] OR antidiabetic*[Title/Abstract]
8. 6 OR 7
9. "Metformin"[Mesh]
10. metformin[Title/Abstract] OR dimethylbiguanidine [Title/Abstract] OR dimethylguanylguanidine [Title/Abstract] OR Glucophage[Title/Abstract]
11. 9 OR 10
12. "Sulfonylurea Compounds"[Mesh]
13. sulfonylurea*[Title/Abstract]
14. 12 OR 13
15. "Thiazolidinediones"[Mesh]
16. Thiazolidinedione*[Title/Abstract] OR Glitazone*[Title/Abstract]
17. 15 OR 16
18. "Insulin"[Mesh]
19. Novolin[Title/Abstract] OR insulin[Title/Abstract]
20. 18 OR 19
21. 8 OR 11 OR 14 OR 17 OR 20
22. "Mortality"[Mesh]
23. "Survival"[Mesh]
24. "Prognosis"[Mesh]
25. prognosis*[Title/Abstract]) OR survival[Title/Abstract]) OR recurrence*[Title/Abstract]) OR mortality[Title/Abstract]) OR predict*[Title/Abstract]) OR outcome*[Title/Abstract]) OR death[Title/Abstract]
26. 21 OR 22 OR 23 OR 24
27. 5 AND 20 AND 25

Supplementary Table 3: Search strategy for Embase (Publication date to 2016/08/17)

1. 'pancreas cancer'/exp
2. (Pancreatic OR Pancreas):ab, ti
3. (Cancer OR tumor OR tumor OR carcinoma OR neoplastic* OR malignant*):ab, ti
4. 2 AND 3
5. 1 OR 4
6. 'antidiabetic agent'/exp
7. (hypoglycemic* OR antihyperglycemic* OR antidiabetic*):ab, ti
8. 6 OR 7
9. 'metformin'/exp
10. (metformin OR dimethylbiguanidine OR dimethylguanylguanidine OR glucophage):ab, ti
11. 9 OR 10
12. 'sulfonylurea derivative'/exp
13. sulfonylurea*:ab, ti
14. 12 OR 13
15. '2,4 thiazolidinedione derivative'/exp
16. (thiazolidinedione* OR glitazone*):ab, ti
17. 15 OR 16
18. 'insulin'/exp
19. (novolin OR insulin):ab, ti
20. 18 OR 19
21. 8 OR 11 OR 14 OR 17 OR 20
22. 'mortality'/exp
23. 'survival'/exp
24. 'cancer prognosis'/exp
25. (prognosis* OR survival OR recurrence* OR mortality OR predict* OR outcome* OR death):ab, ti
26. 22 OR 23 OR 24 OR 25
27. 5 AND 21 AND 26

Supplementary Table 4: Selection procedure of included and excluded studies

	Reference list no.
Studies included in the meta-analysis (n = 14)	1-14
Studies excluded from meta-analysis, and reasons are listed below (n = 28)	15-42
Reason for exclusion	
Publication with cases included in a selected study (n =7)	15-21
No prognostic outcomes recorded (n =3)	22-24
Letters, comments, reviews or meta-analyses (n =12)	25-36
No sufficient data for analysis (n =6)	37-42
